# May the Completion Lead to Conservation: Impact of Partial and Complete Intraoperative Ultrasound Application on Resection Margin Management During Breast-Conserving Surgery for Invasive Breast Cancer: A Retrospective Cohort Study

**DOI:** 10.1245/s10434-025-17822-8

**Published:** 2025-07-24

**Authors:** Oliver Laszlo Stari, Csaba Torok, Petra Otilia Gorog, Balazs Kovacs, Mate Csucska, Beata Kovacs, Bela Markus, Agnes Janovszky, Zoltan Loderer

**Affiliations:** 1 Department of General, Vascular- and Plastic Surgery, Markusovszky Teaching Hospital of Vas County, Szombathely, Hungary; 2 Department of Central Radiology, Markusovszky Teaching Hospital of Vas County, Szombathely, Hungary; 3 Department of Pathology, Markusovszky Teaching Hospital of Vas County, Szombathely, Hungary; 4 Department of Oral and Maxillofacial Surgery, University of Szeged, Szeged, Hungary

**Keywords:** Breast-conserving surgery, Breast cancer, Intraoperative ultrasound, Resection margin

## Abstract

**Background:**

This study aimed to enhance resection margin management during breast-conserving surgery by applying intraoperative ultrasound (IOUS) together with palpation or wire guidance.

**Methods:**

This single-center retrospective observational cohort study was conducted between January 2022 and December 2024. Data were collected from patients with a diagnosis of invasive breast cancer who were treated with lumpectomy. Surgical treatment varied based on the extent of IOUS application. Outcomes included occurrences of positive resection margins (PRM), close resection margins with widths smaller than 1 mm (PCRM<1 mm), and close resection margins with widths smaller than 2 mm (PCRM<2mm). To investigate the extent of breast tissue removal, closest margin widths (CMWs) were compared.

**Results:**

In this study, IOUS was completely used in 72 cases and partially applied in 58 cases, whereas 176 patients underwent standard therapy without IOUS. The PRM rates were 1.4 % for complete IOUS, 5.2 % for partial IOUS, and 13.1 % for standard therapy. The corresponding PCRM<1mm rates were 5.6 %, 17.2 %, and 23.3 %, whereas the corresponding PCRM<2mm rates were 15.3 %, 27.6 %, and 28.4 %. The extent of IOUS application was significantly associated with a reduction in PRM (*p* = 0.007) and PCRM<1mm (*p* = 0.004). Multivariate analysis identified complete IOUS as an independent protective factor against PRM (adjusted odds ratio [aOR], 0.06; 95 % confidence interval [CI], 0.01–0.50; *p* = 0.009), PCRM<1mm (aOR, 0.13; 95 % CI, 0.04–0.41; *p* < 0.001), and PCRM<2mm (aOR, 0.32; 95 % CI, 0.14–0.74; *p* = 0.008) compared with standard therapy. The CMW was similar across study groups (*p* = 0.331).

**Conclusion:**

In breast-conserving surgery for invasive breast cancer, IOUS enhanced resection margin management. The complete application of IOUS may be essential for reducing the rate of close resection margins during IOUS-guided lumpectomy.

Breast-conserving surgery is the preferred surgical treatment for early-stage breast cancer, provided the margins of resected specimens are free of tumor.^1,2^ For decades after the introduction of partial mastectomy, there was no consensus on the definition of a tumor-free resection margin.^3^

In 2014, the Society of Surgical Oncology and the American Society for Radiation Oncology published a consensus guideline recommending a negative resection margin definition of “no ink on the surface of invasive carcinoma or ductal carcinoma *in situ.*”^5^ This consensus led to a decrease in reoperation rate after breast-conserving surgery.^6–8^

A meta-analysis published in 2022 identified a resection margin width smaller than 2 mm as a risk factor for both local and distant recurrence of breast cancer.^9^ After Bundred’s publication, several authors suggested that a minimum resection margin width of 1 mm should be achieved in the treatment of invasive breast cancer.^9,10^ However, modifying the “no ink on tumor” negative margin definition most likely leads to an increase in reoperation rate.^6–8^^)^ Currently, the reoperation rate after breast-conserving surgery ranges from 14.9 to 21.1 %.^11^

Margin management during breast-conserving surgery can be enhanced through various methods.^12–14^ Real-time intraoperative ultrasound (IOUS) in oncologic surgery was introduced in the late 1970s.^15^ In breast surgery, this method was first implemented by Rifkin.^16^ Initial studies focused on the localization capabilities of IOUS for non-palpable breast lesions.^16–19^ During the following decades, randomized trials and cohort studies reported a low reoperation rate and favorable aesthetic outcome after IOUS-guided lumpectomy.^19–27^ Increasing evidence shows that IOUS is an effective tool to enhance intraoperative margin management.^19–26^

Despite the availability of ultrasound devices, IOUS remains underutilized in breast surgery.^23,24^ A lack of standardization in the extent of IOUS application persists.^19^ The number of studies examining the impact of IOUS on resection margin management remains limited, particularly in relation to studies that consider close resection margins as well.

We aimed to assess the impact of partial and complete intraoperative ultrasound application on the likelihood of positive or close resection margins during breast-conserving surgery for invasive breast cancer.

## Method

### Study Design

An institutional review board-approved retrospective observational cohort study was conducted at a teaching hospital in Hungary. The study period spanned from 1 January 2022 to 31 December 2024. The study enrolled 302 patients and involved 306 cases.

### Participants

The inclusion criteria specified female patients age 18 years or older, primary breast-conserving surgery performed during the study period, and a histopathologically confirmed diagnosis of invasive breast cancer. The study excluded patients with complete histopathologic regression after neoadjuvant treatment. The exclusion criterion also ruled out an undefined resection margin status due to incomplete data.

### Study Groups

Study groups were categorized based on the extent of IOUS application. Patients were placed in the complete IOUS group if complete IOUS guidance was applied. Those in the partial IOUS group underwent surgery with partial IOUS application. Patients were assigned to the standard therapy group if IOUS was not used (see the Surgical Therapy section)

### Surgical Therapy

**Fig. 1:**
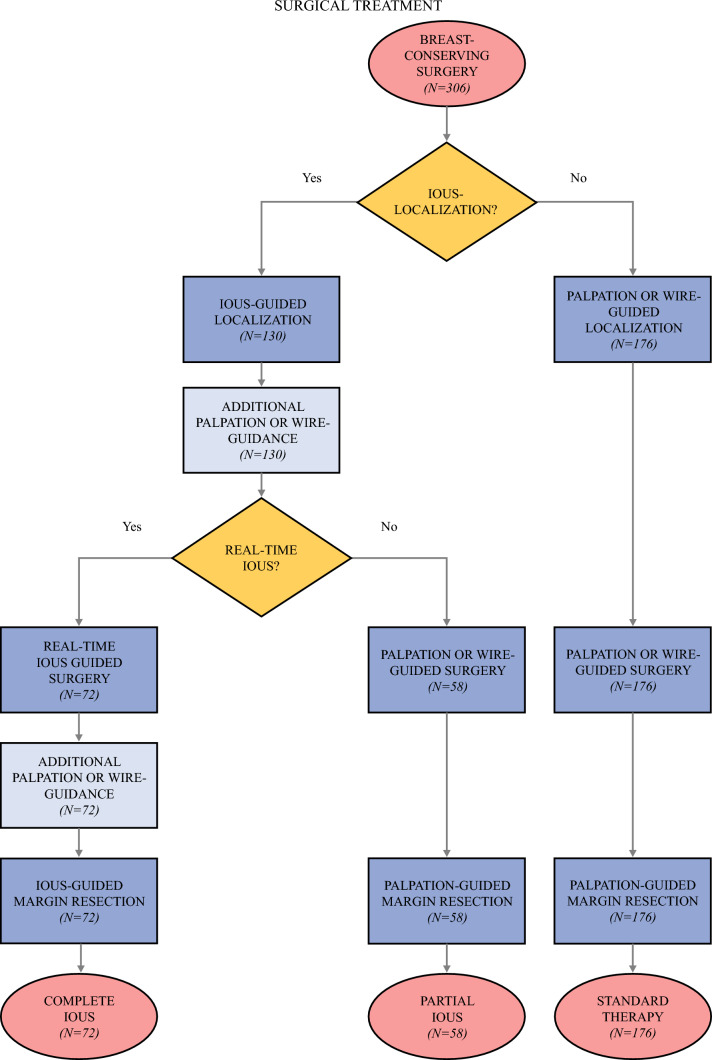
Flowchart of surgical therapy and study grouping. IOUS, intraoperative ultrasound

*Standard Therapy.* During standard surgical therapy, wide local excision was performed. In case of a palpable lesion, the surgery was guided by palpation. For non-palpable lesions, wire-guided localization was used. Removal of the non-palpable lesions was confirmed using intraoperative specimen mammography. Selective margin resection was performed based on the physical examination of the specimen (Fig. 1).

*2.5. Partial Intraoperative Ultrasound Application.* For partial IOUS application, the first step was to locate the malignant lesion and determine its perpendicular extension using IOUS in the operating room. During IOUS-guided localization, distance measurements were performed, and the projection of the tumor was marked on the skin with a marker pen. After the incision, surgery and selective margin resection were performed according to standard therapy protocol. In addition to palpation or specimen mammography, removal of the lesion also was confirmed by *ex vivo* IOUS in the operating room (Fig. 1).

*Complete Intraoperative Ultrasound Application.* For complete IOUS application, the first step was the IOUS-guided localization as described earlier. During surgery, real-time ultrasound guidance was used to assess the distance between the resection planes and the lesion. To enhance visualization of the resection planes, tissue ballottement was performed using a surgical instrument. If the resection margin width was deemed insufficient based on ultrasound examination, it was adjusted accordingly. After specimen removal, an *ex vivo* ultrasound examination was performed to assess the superficial, radial, and deep resection margins. If no healthy breast tissue was detectable between the lesion and the resection planes, an ultrasound-guided selective margin resection was performed. Additional palpation or wire guidance also was used during the IOUS-guided procedures (Fig. 1).

### Quality Control

The treatment of the patients followed the recommendations of the Hungarian Breast Cancer Consensus Guideline, 4th edition.^28^ Regarding IOUS lumpectomies, the surgical teams consisted of the same breast surgeons who performed standard therapy. The IOUS procedure was performed by the surgeon, and a Hitachi-Aloka F37 ultrasound system (Hitachi-Aloka Medical, Tokyo, Japan), operating in the factory-preset Surgery mode with an 8-MHz frequency, was used during the procedures.

During the first five partial IOUS procedures and the first five complete IOUS surgeries, a radiologist with a complex breast diagnostic license was present in the operating room. In addition, intraoperative radiologic consultation remained available.

### Histopathologic Processing and Evaluation

The specimens were processed following formalin fixation. Based on surgical suture markings, the resection planes were oriented and stained. After they were embedded paraffin, hematoxylin and eosin-stained sections were prepared using both small and large block techniques. Receptor status was evaluated using immunohistochemistry or *in situ* hybridization. Margin widths were recorded with the following precision:

0.0 to 1.0 mm: recorded to two decimal places

1.0 to 1.9 mm: recorded to one decimal place

≥2.0 mm: recorded in whole millimeters.

Histopathologic processing and analysis were performed in a standardized manner, following the Hungarian Breast Cancer Consensus Guideline, 4th edition.^29^

### Variables

*Treatment Variable.* The treatment variable was expressed as the “extent of IOUS application” and categorized as a categorical variable with three nominal categories. These categories corresponded to the study groups.

*Outcome Variables.* The outcome variables also were expressed as the “occurrence of positive or close resection margins.” The outcome variables were binary categorical variables. This study included three distinct outcome variables, defined as follows:

1. Positive resection margin (PRM): the resection margin was considered a positive resection margin if the ink staining was presented on the surface of the invasive tumor or ductal carcinoma *in situ* (DCIS) component during histopathologic evaluation.
2. Positive or close resection margin with a margin width below 1 mm (PCRM<1mm): the resection margin was considered a “positive or close resection margin with a margin width below 1 mm” if the closest distance between the resection margin and the invasive tumor or DCIS component was smaller than 1 mm during histopathologic evaluation.
3. Positive or close resection margin with a margin width below 2 mm (PCRM<2mm): the resection margin was considered a “positive or close resection margin with a margin width below 2 mm” if the closest distance between the resection margin and the invasive breast cancer or DCIS component was smaller than 2 mm during histopathologic evaluation.

In this study, the deep resection margin was not considered a close resection margin if the pectoral fascia was removed. A margin also was classified as PCRM<1mm or PCRM<2mm in cases in which a positive resection margin (PRM) occurred.

*Control Variables.* Control variables were based on closest margin width (CMW), defined as the closest distance between the resection margin and the invasive tumor or DCIS component, measured in millimeters. In cases of a PRM, the CMW was defined as 0.0 mm. The variables “CMW before selective margin resection” and “CMW after selective margin resection” were continuous variables, whereas “CMW above 10 mm” was a binary categorical variable. The variable CMW above 10 mm was categorized after selective margin resection.

### Predictors

Predictors were binary categorical variables selected via the following inclusion criteria: DCIS component, non-palpable lesion, multifocality, lobular type, larger tumor size (T2 or T3), human epidermal growth factor receptor 2 expression, hormone receptor expression, age younger than 60 years, higher grade (grade 2 or 3), neoadjuvant therapy, and lymphovascular invasion. Predictors were chosen based on clinical experience and the review of previous cohort studies of Cauhan^30^ and van Deurzen.^31^

#### Confounding

The proximity of the malignant lesion to the deep resection margin was considered a confounding factor. To reduce confounding, the deep resection margin was not considered a close margin if the pectoral fascia was removed. Additionally, adjusted statistical analyses were performed to further mitigate confounding effects.

#### Missing Data

Instead of the most frequently applied five-category tumor localization system in the literature, this study initially included nine possible tumor localization categories. To ensure data comparability, tumor localizations were re-evaluated by the same radiologist who conducted the primary assessment. During histopathologic evaluation, a small proportion of resection margins “exceeding one centimeter in width” were recorded. In such cases, a uniform value of 12 mm was assigned.

## Statistical Analysis

Continuous variables were expressed as median and interquartile range, whereas categorical variables were expressed as proportion and frequency. Comparisons of continuous variables were performed using the Kruskal-Wallis test, whereas categorical variables were analyzed using the chi-square test or Fisher’s exact test. All statistical tests were two-sided, with a 95 % confidence interval (CI), and a *p* value lower than 0.050 was considered statistically significant.

Multivariate logistic regression analyses were performed to evaluate the impact of surgical treatment on the occurrence of positive or close resection margins, with the standard therapy category serving as the indicator. The model selection procedure for the multivariate analyses followed the approach proposed by Chowdhury and Turin.^32^ Variance inflation factors (VIFs) were used to assess multicollinearity among predictors, with a VIF value greater than 1.5 indicating multicollinearity. Backward elimination was applied with a cutoff *p* value lower than 0.250. Models with up to three, six, and seven covariates were considered for PRM, PCRM<1mm, and PCRM<2mm, respectively. Small-sample corrected Akaike Information Criterion (AICc) was used to differentiate among potential models describing the relationship between predictors and outcome variables. During AICc model selection, models with more covariates were selected if they had a significantly lower AICc value. A difference in AICc values was considered significant when it exceeded 2.0. The results of the multivariate analyses are reported as adjusted odds ratio (aOR), 95 % confidence interval (CI), and *p* value. Data analysis was performed using IBM SPSS Statistics, version 30 (IBM Corp., Armonk, NY, USA).

## Results

### Patient and Tumor Characteristics

In 72 cases, IOUS was completely used, and in 58 cases it was partially applied, whereas 176 patients underwent standard therapy without IOUS. No patients were excluded from the study. A comparison of the study groups showed no statistically significant differences in patients’ demographics or in the clinicopathologic characteristics of the lesions. Similarly, no significant differences were observed among the study groups concerning predictors. Missing data did not affect the comparability of the study groups. Descriptive statistics are summarized in Table 1.

**Table 1:** Categorical variables expressed as column percentages and frequency and continuous variables expressed as median and interquartile range^a^

Study groups	Complete IOUS	Partial IOUS	Standard therapy	p
N	72	58	176	
Age	65.0(55.0-72.0)	63.5(56.0-70.0)	64.0(53.0-73.0)	.931
Age below 60 years	33.3(24)	32.8(19)	39.2(69)	.551
Tumor size	1.6(1.1-2.1)	1.9(1.3-2.5)	1.6(1.1-2.1)	.244
Non-palpable	29.2(21)	25.9(15)	26.1(46)	.877
Tl	69.4(50)	55.2(32)	69.3(122)	.118
T2	29.2(21)	44.8(26)	29.5(52)	.078
T3	1.4(1)	0.0(0)	1.1(2)	1.000*
T2 or 3	30.6(22)	44.8(26)	30.7(54)	.118
Lymphovascular invasion	38.9(28)	22.4(13)	30.7(54)	.124
Lymphnode involvement	31.9(23)	29.3(17)	23.9(42)	.391
Upper-outer quadrant	48.6(35)	43.1(25)	51.7(91)	.524
Upper-inner quadrant	23.6(17)	24.1(14)	19.9(35)	.738
Lower-outer quadrant	13.9(10)	13.8(8)	13.1(23)	1.000
Lower-inner quadrant	5.6(4)	12.1(7)	7.4(13)	.364
Central	8.3(6)	6.9(4)	8.0(14)	1.000
Multifocality	15.3(11)	8.6(5)	10.2(18)	.405
DCIS component	31.9(23)	37.9(22)	35.2(62)	.775
Non-specific type	69.4(50)	79.3(46)	77.8(137)	.305
Lobular type	15.3(11)	8.6(5)	9.1(16)	.323
Tubular type	11.1(8)	3.4(2)	5.1(9)	.174*
Mucinose type	2.8(2)	6.9(4)	5.1(9)	.547*
Other type	1.4(1)	1.7(1)	2.8(5)	.877*
Grade 1	43.1(31)	34.5(20)	45.5(80)	.344
Grade 2	45.8(33)	53.4(31)	43.8(77)	.441
Grade 3	11.1(8)	12.1(7)	10.8(19)	.968
Grade 2 or 3	56.9(41)	65.5(38)	54.5(96)	.344
HR expression	83.3(60)	86.2(50)	86.9(153)	.808
HER2 expression	12.5(9)	10.3(6)	14.8(26)	.680
Neoadjuvant therapy	2.8(2)	5.2(3)	4.0(7)	.786*

IOUS, intraoperative ultrasound; DCIS, ductal carcinoma *in situ;* HR, hormone receptor; HER2, human epidermal growth factor receptor 2

^a^Categorical variables were compared using the chi-square test or *Fisher’s exact test, and continuous variables were compared using the Kruskal-Wallis test.

### Outcome

The PRM rate was 1.4 % (1) for complete IOUS, 5.2 % (3) for partial IOUS, and 13.1 % (23) for standard therapy. The corresponding rates were 5.6 % (4), 17.2 % (10) and 23.3 % (41) for PCRM<1mm and 15.3 % (11), 27.6 % (16) and 28.4 % (50) for PCRM<2mm.

Chi-square tests of independence were performed to examine the association between the extent of IOUS application and the occurrence of positive or close resection margins. The analysis indicated a significant association between the extent of IOUS application and PRM (χ^2^ [2, *n* = 306] = 9.85; *p* = 0.007). Similarly, the association between the extent of IOUS application and PCRM<1mm was significant (χ^2^ [2, *n* = 306] = 10.93; *p* = 0.004). However, no significant association was observed between the extent of IOUS application and PCRM<2mm (χ^2^ [2, *n* = 306] = 4.90; *p* = 0.089).

Multivariate logistic regression analyses were performed to assess the impact of IOUS application on the likelihood of positive or close resection margin occurrence. The model selection procedure is presented in Table 2. Variance inflation factors indicated no significant multicollinearity among predictors (range, 1.06–1.30). For PRM, the AICc procedure demonstrated that the model with a DCIS component and multifocality (AICc, 123.5) provided a better fit to the data than those with alternative predictors (AICc range, 129.4–145.7). For PCRM<1mm, the AICc procedure demonstrated that the model with the DCIS component, multifocality, larger tumor size, and higher grade (AICc, 194.4) provided a better fit to the data than those with alternative predictors (AICc range, 194.5–211.5). Backward elimination identified DCIS component, multifocality, and larger tumor size as considerable covariates for PCRM<2mm. For the number of covariates within the predetermined range, the AICc procedure was not performed for PCRM<2mm.

**Table 2:** Model selection procedure for the multivariate analyses

**STEP 1**	**Outcome**	**PRM and PCRM<1 mm and PCRM<2 mm**					
Candidate variables^a^	Candidate Variables	DCIS HR expression	Non-palpable Age<60 years	Multifocality Grade 2 or 3	Lobular type LI	T2 or 3 NAT	HER2
**STEP 2**	**Outcome**	**PRM**		**PCRM<l mm**		**PCRM<2 mm**	
Variables to inculde in the models^b^	Selected variables	DCIS HER2	Multifocality	DCIS T2 or 3 LI	Multifocality Grade 2 or 3	DCIS Grade 2 or 3	Multifocality
**STEP 3**	**Outcome**	**PRM**					
Model selection^c^	Models	Model 1	Model 2	Model 3	Model 4	Model5	Model 6
		IOUS	IOUS	IOUS	IOUS	IOUS	IOUS
		DCIS	-	-	DCIS	DCIS	-
	Covariates	-	Multifocality	-	Multifocality	-	Multifocality
		-	-	HER2	-	HER2	HER2
	AlCc value	137.4	130.2	145.7	123.5	137.0	129.4
	**Outcome**	**PCRM<l mm**					
	Models	Model 1	Model 2	Model 3	Model 4	Model 5	Model 6
		IOUS	IOUS	IOUS	IOUS	IOUS	IOUS
		DCIS	DCIS	DCIS	DCIS	-	DCIS
	Covariates	Multifocality	Multifocality	Multifocality	-	Multifocality	Multifocality
		T2 or 3	T2 or 3	-	T2 or 3	T2 or 3	T2 or 3
		Grade 2 or 3	-	Grade 2 or 3	Grade 2 or 3	Grade 2 or 3	Grade 2 or 3
		-	LI	LI	LI	LI	LI
	AlCc value	194.4	195.1	195.4	211.5	209.8	194.5

PRM, positive resection margins; PCRM<1mm, close resection margin with a width smaller than 1 mm; PCRM<2mm, close resection margin with a width smaller than 2 mm; DCIS, ductal carcinoma in situ; HER2, human epidermal growth factor receptor 2 expression; HR, hormone receptor; LI, lymphovascular invasion; NAT, neoadjuvant therapy; IOUS, intraoperative ultrasound

^a^Expert opinion and review of the literature

^b^Backward elimination (*p* < 0.25)

^c^Small-sample corrected Akaike Information Criterion (AICc)

The analysis for PRM yielded an adjusted odds ratio (aOR) of 0.06 for complete IOUS (95 % CI, 0.01–0.50; *p* = 0.009). The analysis for PCRM<1mm yielded an aOR of 0.13 for complete IOUS (95 % CI, 0.04–0.41; *p* < 0.001). The analysis for PCRM<2mm yielded an aOR of 0.32 for complete IOUS (95 % CI, 0.14–0.74; *p* = 0.008). Because the upper bounds of all the confidence intervals were below 1.0 for all the outcome variables, we can conclude that a significant negative association exists between complete IOUS application and the occurrence of positive or close resection margins. Additionally, the multivariate analyses indicated no significant association between partial IOUS application and PRM (aOR, 0.33; 95 % CI, 0.09–1.23; *p* = 0.098), PCRM<1mm (aOR, 0.54; 95 % CI, 0.23–1.27; *p* = 0.157), or PCRM<2mm (aOR, 0.80; 95 % CI, 0.38–1.70; *p* = 0.564).

### Selective Margin Resection

Intraoperative selective margin resection was performed in 17 (23.6 %) cases with complete IOUS, whereas it was executed in 5 (8.6 %) cases with partial IOUS and in 5 (2.8 %) cases with standard therapy. Univariate logistic regression analysis indicated that it was significantly less likely to perform selective of margin resection with partial IOUS (OR, 0.31; 95 % CI, 0.11–0.89; *p* = 0.029) or standard therapy (OR, 0.10; 95 % CI, 0.03–0.27; *p* < 0.001) than with complete IOUS.

### Extent of Breast Tissue Removal

**Fig. 2:**
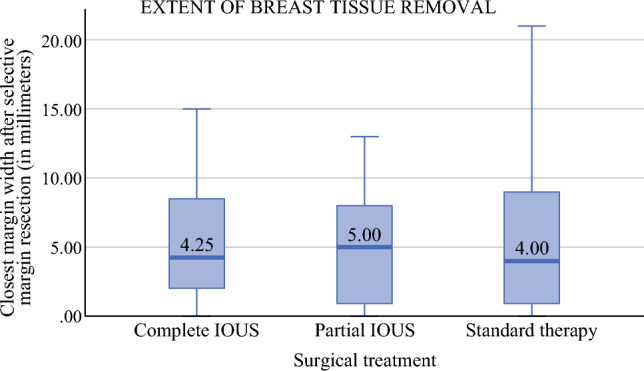
Horizontal lines show the median values; vertical bars display the interquartile range; and vertical lines present the minimum and maximum range. IOUS, intraoperative ultrasound

Before selective margin resection, the median CMW was 3.5 mm (interquartile range [IQR], 1.5–8.0) with complete IOUS, 3.5 mm (IQR, 0.8–8.0) with partial IOUS, and 4.0 mm (IQR, 0.9–8.0) with standard therapy. The median CMW values after selective margin resection were respectively 4.3 mm (IQR, 2.0–8.5), 5.0 mm (IQR, 0.9–8.0), and 4.0 mm (IQR, 0.9–9.0) (Fig. 2). The CMW after selective margin resection exceeded 10 mm in 11 (15.3 %) cases with complete IOUS, in 3 (5.2 %) cases with partial IOUS, and in 24 (13.6 %) cases with standard therapy.

Kruskal-Wallis tests indicated no significant difference across the study groups in CMW before selective margin resection (2, *n* = 306) = 0.45 (*p* = 0.799) and CMW after selective margin resection (2, N=306) = 2.21(*p* = 0.331). A chi-square test of independence found no significant association between the extent of IOUS application and CMW above 10 mm (χ^2^ [2, *n* = 306] = 3.58; *p* = 0.167).

### Characteristics That Make the Localization of the Tumor Challenging

Table 1 presents the rate of tumors with the following characteristics: non-palpable lesions, DCIS component, multifocality, and lobular type. In Table 3, the outcome variables are presented according to the characteristics that make the localization of the tumor challenging. Chi-square tests of independence indicated no significant association between study grouping and the presentation of non-palpable lesions, DCIS component, multifocality, and lobular type (Table 1).

**Table 3:** Characteristics that make the localization of the malignant lesions challenging

Characteristics	Surgical technique	N	PRM	PCRM<l mm	PCRM<2 mm
Non-palpable lesion	Wire-guidance alone	46	6 (13.0)	10 (21.7)	13 (28.3)
	Partial IOUS along with wire-guidance	15	1 (6.7)	2 (13.3)	4 (26.7)
	Complete IOUS along with wire-guidance	21	1 (4.8)	1 (4.8)	3 (14.3)
DCIS component	Palpation or wire-guidance alone	63	14 (22.2)	26 (41.3)	29 (46.0)
	Partial IOUS along with palpation or wire-guidance	22	2 (9.1)	5 (22.7)	8 (36.4)
	Complete IOUS along with palpation or wire-guidance	23	1 (4.3)	2 (8.7)	7 (30.4)
Multifocality	Palpation or wire-guidance alone	18	8 (44.4)	11 (61.1)	13 (72.2)
	Partial IOUS along with palpation or wire-guidance	5	1 (20.0)	2 (40.0)	3 (60.0)
	Complete IOUS along with palpation or wire-guidance	11	1 (9.1)	3 (27.3)	6 (54.5)
Lobular type	Palpation or wire-guidance alone	16	3 (18.8)	4 (25.0)	6 (37.5)
	Partial IOUS along with palpation or wire-guidance	5	1 (20.0)	2 (40.0)	3 (60.0)
	Complete IOUS along with palpation or wire-guidance	11	0 (0.0)	0 (0.0)	1 (9.1)
None of the above	Palpation alone	67	5 (7.5)	9 (13.4)	10 (14.9)
	Partial IOUS along with palpation	21	0 (0.0)	3 (14.3)	5 (23.8)
	Complete IOUS along with palpation	29	0 (0.0)	0 (0.0)	2 (6.9)

Variables expressed as frequency and row percentages; PRM, positive resection margin; PCRM<1mm, close resection margin with a width smaller than 1 mm; PCRM<2mm, close resection margin with a width smaller than 2 mm; IOUS, intraoperative ultrasound; DCIS, ductal carcinoma *in situ*

### Patient Safety

No complications related to intraoperative ultrasound were observed during the study period. The overall complication rate did not differ from the expected rate based on previous clinical experience.

## Discussion

The application of IOUS during breast-conserving surgery has been associated with a low rate of positive resection margins.^12,19^ In the randomized COBALT trial, 3 % of the patients treated with IOUS-guided surgery had tumor-involved resection margins compared with 17 % of the patients treated with palpation-guided surgery, a statistically significant difference (*p* = 0.009).^20^ Similar findings have been reported in multiple other studies.^17–19,21–26^ Our study found a significant association between the extent of IOUS application and PRM, with respective rates of 1.4 %, 5.2 % and 13.1 % across the study groups (*p* = 0.007).

The effect of IOUS on close resection margins has been less extensively studied. Eggemann et al.^33^ reported that the application of IOUS guidance led to a close resection margin rate of 14.3 %. This 2014 prospective cohort study defined close margin as a resection margin width smaller than 1 mm. Regarding our results, the rates of PCRM<1mm were 5.6 % for complete IOUS, 17.2 % for partial IOUS, and 23.3 % for standard therapy. Chi-square testing indicated a significant association between the extent of IOUS application and PCRM<1mm (*p* = 0.004). Multivariate analysis further demonstrated that the application of complete IOUS was an independent protective factor against PCRM<1mm compared with standard therapy (aOR, 0.13; 95 % CI, 0.04–0.41; *p* < 0.001).

In a 2011 retrospective study, Fisher et al.^34^ performed a reoperation if the resection margin width fell short of 2 mm. The statistical analysis showed no difference in reoperation rates between the IOUS-guided and palpation-guided methods (23 % vs 25 %; *p* > 0.05).^31^ We observed similar rates for PCRM<2mm with partial IOUS and standard therapy (27.6 % vs 28.4 %, respectively). It is important to note that the partial IOUS method used in our study closely resembled the technique described by Fisher et al.^34^ The similarity between these findings suggests that the beneficial effect of partial IOUS application may be limited to positive resection margins. However, the adjusted analysis in our study identified complete IOUS application as an independent protective factor against PCRM<2mm compared with standard therapy (aOR, 0.32; 95 % CI, 0.14–0.74; *p* = 0.008).

Additionally, we observed that the application of IOUS led to enhanced resection margin management regardless of the presentation of the following characteristics known for making the localization of the tumor difficult: non-palpable lesions, DCIS component, multifocality, and lobular type (Table 3). A 2019 cohort study compared IOUS and wire-guided surgery for non-palpable lesions.^26^ Esgueva et al.^26^ reported that IOUS guidance showed significantly smaller surgical volumes (*p* = 0.020), a smaller calculated resection ratio (*p* = 0.006), a higher rate of negative margins (*p* = 0.017), and less surgical time (*p* = 0.006) than wire-guidance. In terms of lobular type, we found that the rate of positive or close resection margins tended to be lower with complete IOUS than with standard therapy. Interestingly, the randomized COBALT trial and a 2025 prospective cohort study reported similar observations regarding lobular type. Further, dedicated research is needed to examine the association between IOUS and these tumor characteristics.^20,25^

Considering the extent of breast tissue removal, a 2023 prospective cohort study demonstrated that IOUS-guided surgery resulted in smaller specimen volumes (16.8 vs 24.3 cm^3^; *p* = 0.015) and a significantly higher tumor volume-to-specimen volume ratio than traditional surgery (4.7 % vs 2.9 %; *p* < 0.001).^24^ Ferrucci et al.^24^ achieved these results with a significantly lower rate of positive resection margins in the IOUS-guided group (2.5 % vs 12.5 %; *p* = 0.032).

In our study, we compared the closest margin widths. Regarding CMW after selective margin resection, the analysis indicated no significant difference among complete IOUS, partial IOUS, and standard therapy (median values of 4.3 mm, 5.0 mm, and 4.0 mm, respectively; *p* = 0.331). Moreover, we found no significant association between the extent of IOUS application and the rate of CMW above 10 mm (15.3 %, 5.2 %, and 13.6 % respectively; *p* = 0.137). These findings are particularly interesting on the basis that the performance of selective margin resection was significantly less likely with partial IOUS (OR, 0.31; 95 % CI, 0.11–0.89; *p* = 0.029) and standard therapy (OR, 0.10; 95 % CI, 0.03–0.27; *p* < 0.001) than with complete IOUS. Our results imply that the application of IOUS leads to better margin control than palpation or wire-guidance alone, without the need for extended breast tissue removal.

A key strength of our study was that it analyzed the impact of IOUS based on the extent of its application. Additionally, this study evaluated the effectiveness of IOUS application in relation to both positive and close resection margins.

This study had several limitations. Patient allocation to surgical treatment was not randomized. The generalizability of the findings are limited because the study did not feature cases with pure DCIS, and the analysis was based on data from a single center. The number of cases with non-palpable invasive lesions also was limited, although in our study, this characteristic was not shown to be a risk factor of positive or close margin occurrence. Due to the lack of follow-up data, the postoperative period after discharge could not be included in this study. The limitations inherent to retrospective data collection also were evident, particularly the absence of data on the following important factors: the volume of tissue removed during surgery, patients’ race and ethnicity, aesthetic outcomes, cost-effectiveness, and the time required to perform IOUS.

## Conclusions

The application of intraoperative ultrasound during breast-conserving surgery for invasive breast cancer led to enhanced resection margin management compared with palpation or wire-guidance alone. According to our single-center experience, continuous application of intraoperative ultrasound throughout the procedure may be essential to reduce the rate of close resection margins during intraoperative ultrasound-guided lumpectomy.
